# Knowledge and Practice of Antibiotic Management and Prudent Prescribing among Polish Medical Doctors

**DOI:** 10.3390/ijerph19063739

**Published:** 2022-03-21

**Authors:** Wojciech Stefan Zgliczyński, Jarosław Bartosiński, Olga Maria Rostkowska

**Affiliations:** 1 Department of Lifestyle Medicine, School of Public Health, Center of Postgraduate Medical Education, 01-826 Warsaw, Poland; 2 Department of Anaesthesiology and Intensive Therapy, Independent Public Clinical Hospital No. 4, 20-097 Lublin, Poland; jaroslaw.bartosinski@spsk4.lublin.pl; 3 Department of Transplantation Medicine, Nephrology and Internal Diseases, Medical University of Warsaw, 02-006 Warsaw, Poland

**Keywords:** antibiotic, antimicrobial resistance, hand hygiene, medical doctors, Poland, public health

## Abstract

Antimicrobial resistance (AMR) is an urgent public health issue. The role of medical doctors in proper antibiotic use is crucial. The aim of this study was to evaluate the knowledge and practices of Polish doctors of antimicrobial prescribing and antibiotic resistance. The study group consisted of 504 medical doctors with an average age 32.8 ± 5.9 years, mostly women (65%). The paper questionnaire was developed on the basis of a survey tool developed by the European Centre for Disease Prevention and Control (ECDC) and Public Health England (PHE). According to our study, physicians were aware that: taking antibiotics has side effects, antibiotics cannot be used against viruses, unnecessary use of antibiotics leads to AMR and that healthy people can carry resistant bacteria (each item ≥98% correct responses). Only 47% of respondents knew that the use of antibiotics as growth stimulants in livestock is illegal in the EU. Of the respondents, 98.61% saw the connection between prescribing antibiotics and AMR. However, 65.28% of the respondents reported a lack of appropriate materials on AMR counseling. Nearly 92.5% of participants “never” or “rarely” gave out resources on prudent antibiotic use. Physicians in Poland underestimate the role of hand hygiene in stimulating antibiotic resistance (ABR) (74.4%), while demonstrating satisfying knowledge about antimicrobial use, the clinical application of antimicrobial guidelines and prevention of ABR. However, educational interventions are needed to help lead challenging communication with assertive patients. Appropriate patient resources would be helpful in reaching this goal.

## 1. Introduction

Antimicrobial resistance (AMR) is defined as the reduced or eliminated response of microbes to pharmacological treatment which can be intrinsic to species or obtained through complex mechanisms developed through evolutionary processes [1]. Unjustified use or malpractice in administering antibiotics has contributed greatly to the emergence of the AMR crisis by accelerating the development of antibiotic resistance (ABR) [2]. According to the report by O’Neill, by 2050, an astounding number of 10 million patients per year could die globally due to AMR, causing great loss in social terms but also adding-up to the financial strain already placed on healthcare in the first half of the 21st century by the COVID-19 disease [3,4,5]. Although the “10 million” estimates from O’Neill’s report have been challenged by de Kraker et al. [6], numerous independent experts strongly agree that AMR threatens not only individual human health but also global economies on a large scale [7,8,9]. Even though the ABR emergency has been less spoken of during the SARS-CoV-2 pandemic, the phenomenon continues to evolve. ABR aggravates the condition of coronavirus patients due to life-threatening concomitant bacterial diseases often acquired in the course of the COVID-19 infection [10]. All this makes resistance to antibiotics a recurring point in discussions over Sustainable Development Goals defined by the United Nations [9,11]. According to the World Health Organization, managing AMR remains among the 10 top priorities in providing public health safety [1]. Drivers of the decreased susceptibility of bacteria to antibiotics in healthcare are well examined. Main causes contributing to the spread of resistance include the misuse of antimicrobials in ambulatory care, inaccurate administration in hospitals, ineffective preventive measures against infections (e.g., hand hygiene, vaccination) and a shortage of new pharmacological agents, to name a few [2,12]. Prudent antimicrobial prescribing is of fundamental importance when tackling the challenge of AMR worldwide and locally.

On 18 November 2019, marking the European Antibiotic Awareness Day (EAAD) [13], the European Centre for Disease Prevention and Control (ECDC) and Public Health England (PHE) released a report summarizing the outcomes of a survey on antibiotic use and knowledge among health-care workers (HCW) in 30 EU/EEA countries based on data collected in 2019 [14]. The report was followed by a publication by Ashiru-Oredope et al. (2021) in Eurosurveillance [15] In most European countries, medical doctors are predominantly responsible for diagnosing and administering antimicrobial agents in human medicine, thus remaining the stewards of antibiotic use in healthcare [16]. Scientific research aimed at identifying weak spots, hesitancy, and obstacles which prescribers face in their practice regarding the use of antibiotics is of key importance in ensuring prolonged effectiveness of these medicines.

The purpose of our study was to evaluate the knowledge and practice of doctors in Poland on antimicrobial prescribing and ABR and to complement the pan-European data released in 2019 by the ECDC and PHE.

## 2. Materials and Methods

### 2.1. Study Group

The study group comprised 504 medical doctors participating in specialization courses at the Center of Postgraduate Medical Education in Warsaw, Poland from November 2019 to March 2020, after the ECDC and PHE released their report.

Purpose of the study is consistent with statutory objectives of the Centre of Postgraduate Medical Education (CMKP), which is a Polish public medical university supervised by the Ministry of Health, responsible for conducting, coordinating and controlling postgraduate education of physicians and other selected medical professionals throughout Poland (Act of 13 September 2018 on the Medical Centre for Postgraduate Education (Journal of Laws of 2018, item 2024)).

The presented study is not considered a medical experiment, so according to Polish law, ethical approval is not required (Act of 5 December 1996 on professions of physicians and dentist (Journal of Laws of 2021, item 790 as amended)). The survey used in this study was in line with the ethical standards of the institutional bioethical commission and the Declaration of Helsinki (1964). Although participation in the study was anonymous and voluntary, each surveyed participant expressed consent by filling in the survey.

### 2.2. Survey Questionnaire

The research tool was a self-administered paper questionnaire prepared on the basis of a Polish translation of the validated tool used by the ECDC and PHE [14]. ECDC and PHE used a survey which was made available online in all EU/EEA languages from 29 January 2019 till 1 March 2019, after which the questionnaire was taken down from the website [17]. Since our research was to be launched at a later date, we issued a request in August 2019 to ECDC for the original questionnaire in Polish to be used for the purpose of research after 19 November 2019.

The questionnaire included basic characteristics of the respondent (gender, age, place of residence), professional characteristics (main place of work, type and stage of specialization), multiple choice questions, questions testing antibiotic knowledge using a true or false answer, and statements assessing attitudes and behaviors towards antibiotics by using a 5-point Likert scale from strongly agree to strongly disagree.

The questionnaire was piloted on a sample of 15 respondents working as medical professionals at the School of Public Health, Centre of Postgraduate Medical Education in Warsaw, Poland. As a result of the pilot study, some questions were modified.

In Poland, each physician completing specialty training would attend a course in public health. Participation in this course is compulsory. Therefore, participants on the courses represented all medical specialties, from all regions and healthcare institutions all over Poland.

### 2.3. Calculation of the Antibiotic Knowledge Score

Respondents’ knowledge was assessed in nine questions concerning antibiotic use and resistance.

Value 1 was assigned to each correct answer, and value 0 to each incorrect answer. Knowledge score was constructed as a sum of scores from all the answers. In total, it was possible to obtain a minimum of zero points and a maximum of nine points. Thus, the antibiotic knowledge score had a positive direction; the higher the score, the better the knowledge of antibiotics.

### 2.4. Statistical Methods

The statistical analyses were performed using STATISTICA 13.1 software (STATSOFT, Kraków, Poland). The mean (M) and standard deviation (SD) were estimated for numerical variables, as well as absolute numbers (n) and percentage (%) of the occurrence of items for categorical variables.

Pearson’s chi-square test was used to investigate an association between two categorical variables. Variables in Likert scale or in other ordinal scale and numerical variables were correlated to each other using Spearman’s correlation coefficient. Student’s *t* test was used to compare one variable in the Likert scale between two groups (men vs. women, specialization completed vs. ongoing, surgical vs. non-surgical specialization, clinical hospital vs. other places of work, living in cities vs. towns or villages).

The significance level was assumed to be *p* < 0.05.

## 3. Results

### 3.1. Characteristics of the Study Group

The demographic and professional characteristics of the study group are presented in Table 1.

A total of 504 correctly completed questionnaires filled by medical doctors were included in the study. The participants were aged 25–59 years, 32.8 ± 5.9 years on average, mostly women (65%). The response rate was 81%. Analyzing in 10-year age groups, 30–39-year-old respondents predominated in the study group (58%), followed by 20–29-year-olds (30%), whereas older respondents constituted 11% of the study group. The majority of respondents lived in cities with over 500 thousand residents (54%). The questionnaire was delivered in paper form to physicians attending public health specialization courses at the CMKP.

Respondents’ length of work as medical doctors was up to 39 years, with an average of 5.1 ± 5.8 years. Analyzing groups of work seniority, respondents who had been working 1–3 years predominated in the study (58%), followed by respondents who had been working 4–7 years (21%), while 17% of respondents had worked for at least 8 years as medical doctors.

The main place of work for most respondents was a public hospital (42%), followed by a clinical hospital (40%). Participants represented all 77 medical specialties recognized in Poland. A total of 81% respondents had no specialization (ongoing), while 15% of respondents had already completed at least one specialization. The majority of respondents already completed or were during training in non-surgical specialization (80%), while 16% were in a surgical one.

### 3.2. Antibiotics and Antibiotic Resistance Knowledge

Table 2 presents nine items concerning antibiotic knowledge. All respondents correctly answered that “Taking antibiotics has associated side effects or risks such as diarrhea, colitis, allergy”. Almost all disagreed with statements “Antibiotics are effective against viruses” and “Antibiotics are effective against cold and flu” (503 and 502 out of 504 respondents, respectively). The majority of respondents gave correct answers that “Unnecessary use of antibiotics makes them ineffective” and “Healthy people can carry antibiotic-resistant bacteria” (97.62% correct answers on each item).

Furthermore, the majority of respondents correctly answered the following items: “Antibiotic-resistant bacteria can spread from person to person” (94.84%) and “Every person treated with antibiotics is at an increased risk of antibiotic-resistant infection” (89.88%), followed by “The phenomenon of drug resistance in the context of infectious diseases only occurs with bacteria (not e.g., viruses)” (80.95%). An item which received least correct answers was “The use of antibiotics to stimulate growth in farm animals is legal in the EU” (47.02%).

**Figure 1:**
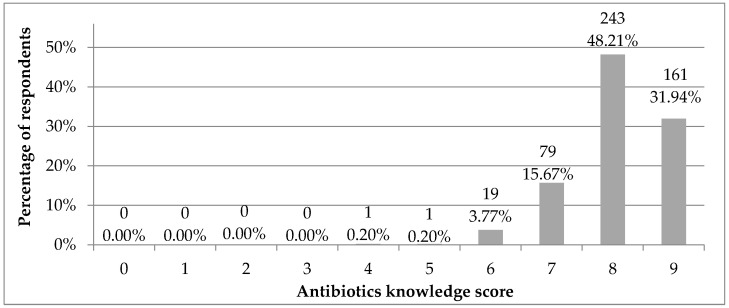
Antibiotics knowledge score (number of correct answers) in the study group (*N* = 504). Scale from 0—no correct answer to 9—all the correct answers. Results are presented as the number and percentage of respondents.

Based on nine items regarding antibiotic knowledge from Table 2, the antibiotics knowledge score was calculated as the number of correct answers that could oscillate from zero—no correct answer, to nine—all the correct answers (Figure 1). Most respondents (48%) gave eight out of nine correct answers followed by 32% respondents with all correct answers. Approximately 16% of respondents chose seven correct answers, six correct answers were chosen by 4% respondents, while four or five correct answers by one respondent each. None of the respondents gave zero, one, two or three correct answers.

The antibiotic knowledge scores did not correlate with gender, living or not in a large city, specialization status, type of specialization, working or not in a clinical hospital, nor with age and job seniority (due to too low values of correlation coefficients).

**Figure 2:**
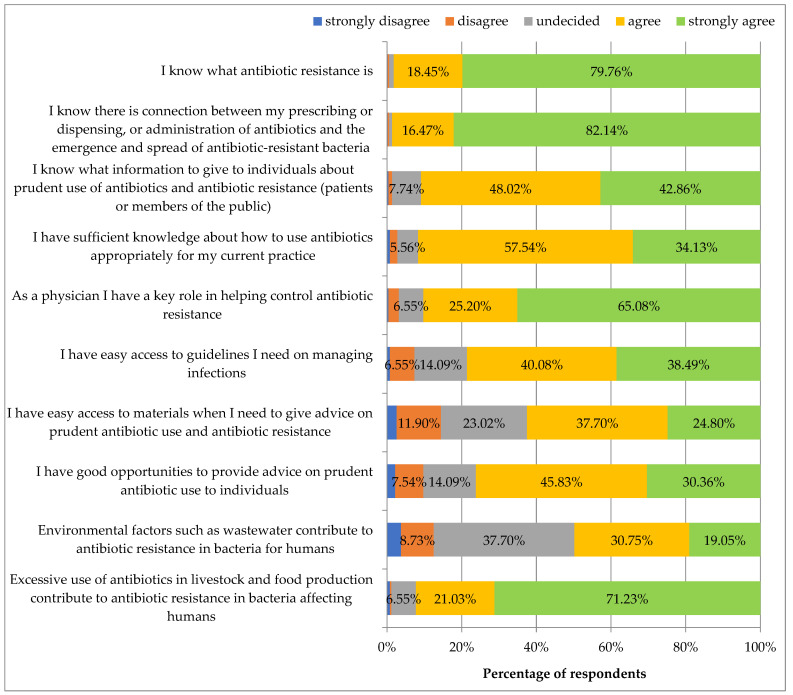
Do you agree or disagree with the following statements regarding antibiotic knowledge?

Figure 2 presents some statements regarding antibiotic knowledge The respondents agreed most with the statements: “I know what antibiotic resistance is” (79.76% strongly agreed and 18.45% agreed) and “I know there is connection between my prescribing or dispensing or administering antibiotics and the emergence and spread of antibiotic-resistant bacteria” (82.14% strongly agreed and 16.47% agreed), followed by “Excessive use of antibiotics in livestock and food production contribute to antibiotic resistance in bacteria affecting humans” (71.23% strongly agreed and 21.03% agreed) and “As a physician, I have a key role in helping control antibiotic resistance” (65.08% strongly agreed and 25.20% agreed. The respondents agreed less with the following statements: “I have good opportunities to provide advice on prudent antibiotic use to individuals”, “I have easy access to guidelines I need on managing infections”, “I have sufficient knowledge about how to use antibiotics appropriately for my current practice” and “I know what information to give to individuals about prudent use of antibiotics and antibiotic resistance (patients or members of the public)”.

The respondents least agreed with the following statements: “I have easy access to materials when I need to give advice on prudent antibiotic use and antibiotic resistance” and “Environmental factors such as wastewater contribute to antibiotic resistance in bacteria for humans”.

Female respondents perceived their role in helping control ABR as key significantly more often than men (4.9 ± 0.4 vs. 4.6 ± 0.6), (*p* = 0.010).

Respondents working in clinical hospitals perceived their access to patient-targeted educational materials on prudent antibiotic use and ABR as less easy (3.5 ± 1.1) than those not working in clinics (3.9 ± 1.1), (*p* = 0.027).

The data on antibiotic subjective knowledge presented in Figure 2 did not correlate with: specialization status, type of specialization, or living in a large city (*p* > 0.05) as well as with age and job seniority.

**Figure 3:**
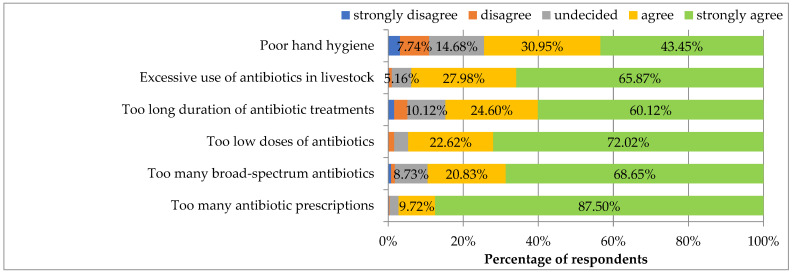
Importance of individual factors for the development of antibiotic resistance.

Figure 3 presents the perceived importance of selected factors for the development of ABR. The respondents agreed significantly with all the factors The respondents considered poor hand hygiene as the least important for the development of ABR, whereas the most important for the development of ABR was too many antibiotic prescriptions followed by too low doses of antibiotics, excessive use of antibiotics in livestock and too many broad-spectrum antibiotics. Too long duration of antibiotic treatments was in the middle of the ranking of factors contributing to ABR.

Too long duration of antibiotic treatments and too many broad-spectrum antibiotics were considered more important for the development of antibiotic resistance in the females’ opinion than in the opinion of men (*p* = 0.001 and 0.005, respectively).

The importance of factors responsible for antibiotic resistance did not correlate with specialization status, type of specialization, working in a clinical hospital or living in a large city (*p* > 0.05) or with age and job seniority (too low values of correlation coefficients).

### 3.3. Attitudes and Behaviors on Antibiotic Use and Antibiotic Resistance

Of the respondents, 17.26% prescribed or administered antibiotics to their patients more often than once a day, 9.56%—once a day, 9.36%—4–6 times per week, 28.27%—2–3 times per week, 16.01%—once a week, 11.85%—once or twice a month, 6.42%—less than once a week, 1.46%—never.

The frequency of prescribing or administering antibiotics to patients did not correlate with the respondents’ gender (*p* = 0.073), age (*p* = 0.308), job seniority (*p* = 0.463), specialization status (*p* = 0.542), type of specialization (*p*-0.402), working or not in a clinical hospital (*p* = 0.280), or living in a large city (*p* = 0.306).

Respondents used the following strategies for antibiotic prescription: the most common—patient education (73.02%), less common—delayed/back-up prescribing (delayed prescribing is the method by which a health worker issues a prescription for a patient to be used at a later date, if there is no improvement), (34.72%) and the least common—new patient consultation (11.90%). Male respondents marked new patient consultation more often (17.06% vs. 9.17%, *p* = 0.010). The prevalence of the three above-mentioned strategies for rational antibiotic prescribing did not correlate with age, job seniority, specialization status, type of specialization, working or not in a clinical hospital, or living in a large city (*p* > 0.05).

Only 4.56% of respondents were able to provide their patients with advice or resources as needed. The most common reasons were: the lack of available materials (indicated by 65.28% of respondents) and insufficient time (58.73%). Furthermore, 39.29% of respondents indicated that a patient was not interested in information, 17.86% that a patient did not need such information, 5.16% were not sure what advice to provide, 4.96% indicated difficulty in getting patients to understand the diagnosis, 3.77% indicated language barriers. Lack of materials available was significantly more prevalent among respondents living in large cities rather than villages or towns (69.94% vs. 56.29%), (*p* = 0.003); with ongoing specialization rather than a completed one (67.91% vs. 56.96%), (*p* = 0.0.44) and in younger respondents (*p* = 0.010). The prevalence of other reasons why physicians were unable to provide resources to their patients did not correlate with gender, job seniority, type of specialization or working in a clinical hospital (*p* > 0.05).

**Figure 4:**
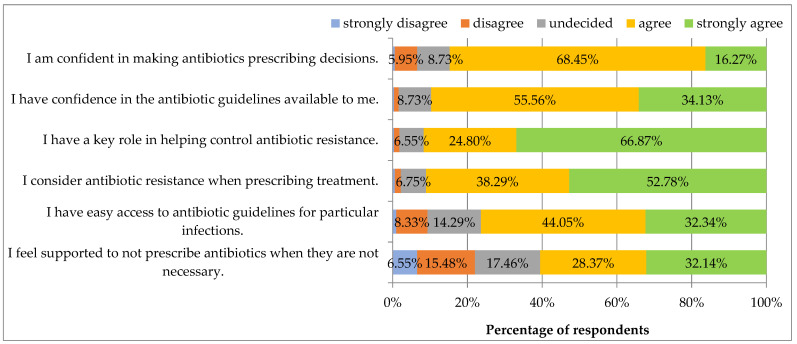
To what extent do you agree or disagree with the following statements?

Figure 4 presents the statements regarding attitudes and behaviors on antibiotic use and antibiotic resistance. All statements received a high percentage of agreement or strong agreement. The respondents mostly agreed that they have a key role in helping control ABR, followed by consideration of ABR when prescribing a treatment, trust in available guidelines, confidence in making prescribing decisions and having easy access to guidelines for particular infections. The fewest respondents agreed with the statement that they felt supported in not prescribing antibiotics when they were not necessary.

Respondents were more confident in making antibiotic prescribing decisions if they did not work in a clinical hospital (*p* = 0.046). The respondents perceived their key role in helping control ABR as more important if they were without specialization (*p* = 0.040). Female respondents felt more confident in not prescribing antibiotics when not necessary (*p* = 0.006). The statements regarding some attitudes and behaviors on antibiotic use and antibiotic resistance presented in Figure 4 did not correlate with the type of specialization or living in a large city (*p* > 0.05) or with age and job seniority (too low values of correlation coefficients).

**Figure 5:**
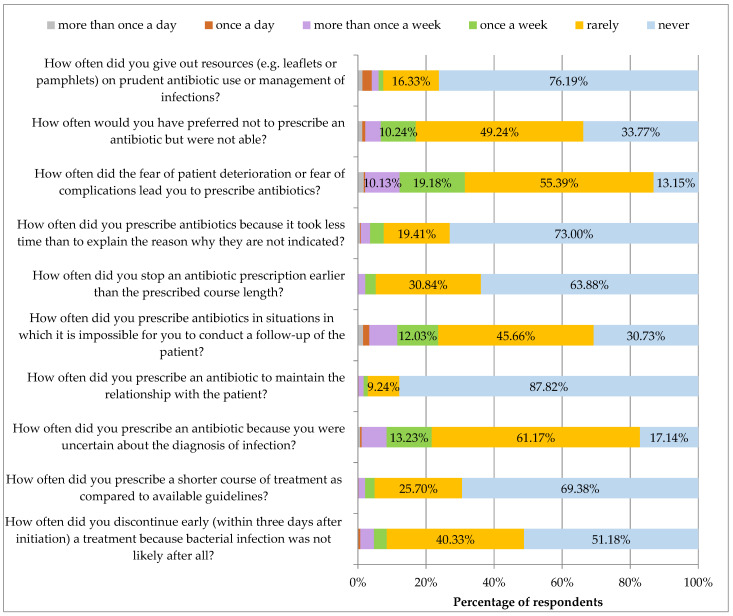
Selected practices regarding antibiotic use and prescribing. Percentages were calculated from the answers given.

Figure 5 presents respondents’ practices regarding antibiotic use. Over 90% of respondents claimed that they did not give out resources (e.g., leaflets or pamphlets) on prudent antibiotic use or management of infections (76.19% never and 16.33% rarely).

More than one tenth of respondents declared that they had prescribed antibiotics when they had preferred not to do so. Almost one third of respondents prescribed antibiotics due to fear of the patient’s condition deteriorating or health complications. Furthermore, one fifth of respondents claimed that they prescribed antibiotics because they were uncertain about the diagnosis of infection. One quarter of respondents declared that they ordered antibiotics in situations in which it was impossible to follow-up on the patient

On the other hand, more than 90% of respondents would never or rarely stop an antibiotic treatment earlier than the due course length, prescribe a course of treatment shorter than in the guidelines or discontinue treatment earlier if a bacterial infection was not likely after all. Almost three-quarters of respondents claimed that they never prescribed antibiotics because it took less time than to explain the reason why they were not indicated. Hardly any respondents had prescribed antibiotics to maintain a relationship with a patient.

## 4. Discussion

The research outcomes presented here are among a few studies which have employed the ECDC and PHE survey model to collect information on the antibiotic knowledge and prescribing practices of HCWs in a specific country and among a selected group of professionals. Our work delivers a Polish complement to the ECDC/PHE data presented in the 2019 report, focusing on doctors.

Regarding knowledge questions, our results mirror those presented by the ECDC/PHE with the most accurate answers being given for “Antibiotics are effective against viruses”, “Antibiotics are effective against cold and flu” and “Taking antibiotics has associated side effects or risks such as diarrhea, colitis, allergies” as those questions were answered correctly in our study by at least 99.6% of respondents. Similar scores (91% of correct answers or over) were presented by Italian and Jordanian researchers using the same questionnaire tool among prescribers [14,18,19]. Interestingly, in a recent Swedish nationwide population-based study, antibiotic use was positively associated with proximal colon cancer but inversely with rectal cancer which should be considered when opting for treatment with antibiotics [20]. Conversely, the lowest correct response rate was obtained in the examined populations (Jordan, Europe) as to whether using antibiotics in agriculture to stimulate growth of animals is legal—47% correct answers in our study (“not legal in the EU”), 27% in the ECDC/PHE study (“not legal in the EU”) and 19% in the Jordanian study (“not legal in Jordan”) [14,17]. The Italian study did not provide data on this aspect [18]. The use of antibiotics in European farming as growth promoters has been banned since 2006 and in the beginning of 2022 a new directive comes fully into force further restricting preventive use of antimicrobials in agriculture and ensuring partners from outside Europe comply when trading food products with EU member states [21,22,23]. Based on all surveys results, the awareness of the One Health approach combining human and animal well-being in the context of antibiotics is one that needs strengthening in the medical community across different countries.

Most of our respondents declared that they know what antibiotic resistance is (knowledge score 4.8 ± 0.5 on a 5-point scale). Based on the ECDC/PHE data, 96% of respondents (HCW) know what ABR is, compared to 87% in the study completed in Jordan [14,19]. Similar results were obtained from our study (4.8 ± 0.5), given the awareness of physicians regarding links between their use of antibiotics in patients and the emergence of ABR in bacteria. In the ECDC/PHE study, 92% of respondents were aware that their prescribing, administration and dispensing of antibiotics affects the spread of AMR [14]. In the study of Italian HCWs, over 98% of physicians knew the importance of this interaction [18]. In the study of physicians in Jordan, only 68% agreed or strongly agreed that their prescribing and/or administration of antibiotics had a connection with ABR [19]. Regarding the use of antibiotics in livestock and food production, 89% of HCWs in the ECDC study and less than 60% of physicians in the Jordanian research agreed this could affect bacteria causing infections in humans [14,19]. In our study, a relatively high knowledge score of 4.6 ± 0.7 was declared considering this aspect of antibiotic use (third highest knowledge score, overall).

Regarding factors promoting antimicrobial resistance, most relevant to our respondents were “too many antibiotic prescriptions” and “too low doses of antibiotics” with scores of 97.22% and 94.64% of respondents agreeing, respectively. According to a 2019 literature review by Chokshi et al., suboptimal hospital surveillance over antibiotics administered in clinics and the use of antimicrobials in animal farming are the biggest contributors to AMR in developed countries [24]. Clinical misuse and ambulatory overprescribing are more prevalent in low-income countries [24]. Diverse stakeholders advocate for the One Health approach which promotes joining forces in the responsible use of antibiotics in medicine, agriculture, farming and food processing to halt the spread of AMR [25,26]. The option “Excessive use of antibiotics in livestock” was placed in the middle of the scale in terms of influence on ABR according to our respondents (93.85% of respondents agreed). Interestingly, hand hygiene scored lowest (74.40% of respondents agreed) as a factor impacting ABR. Proper hand hygiene is considered a core element of infection prevention and control by WHO and independent scholars. By decreasing the incidence of infections, hygiene translates into a lower need for antibiotics, especially in hospital care [27,28,29]. During the pandemic of SARS-CoV-2, hand hygiene together with other infection control measures have been reinforced as fundamental prophylaxis means for decelerating the spread of coronavirus [30,31]. This can also favorably impact antimicrobial resistance, according to the analyses presented by Collignon and Beggs, as well as Sharmila Devi [32,33]. However, the long-term impact of the COVID-19 pandemic on AMR has yet to be defined.

Our respondents agreed (90.28% of them) about the key role of physicians in controlling the spread of antimicrobial resistance. Doctors can contribute by encouraging the use of vaccines among patients, reinforcing infection-control procedures, managing antibiotic surveillance, engaging in research on novel therapy agents, promoting good practice of guideline-adherence when prescribing antimicrobials and finally, by educating future generations of health-care workers about AMR [34,35,36]. Infectious disease specialists and general practitioners, especially, are in key positions when discussing prevention and antimicrobial prescribing [34,37]. At the same time, the One Health strategy is gaining momentum where unisono cooperation between medical doctors, veterinarians, agriculture experts and food-chain agents becomes a key approach to combat AMR [38,39,40]. However, this interdisciplinary cooperation requires well-thought out communication and good understanding of the complexities of each field.

Based on the results of our study, it occurred to us that much focus is needed regarding communication with the public on the inappropriateness of prescribing antibiotics when they are not necessary. Many of our respondents claimed that they do not feel confident in declining the demand for an antibiotic prescription even when it is not supported by clinical evidence. This is especially valid in treatment of acute upper respiratory tract infections (ICD-10 codes J00-J06), which in roughly 80% of cases are caused by viruses [41]. Problematic use of antibiotics in the general population in Poland is common, although an optimistic trend in awareness was observed in recent years by Mazińska et al. [42]. However, many patients still expect antibiotics as an assumed optimal cure for their ailment, despite no evidence of a bacterial etiology for their condition [43]. In fact, it is usually the pressure for a quick solution from a patient combined with time-constraints of an ambulatory visit that lead to unjustified antibiotic prescribing [44,45]. In our survey, 27% of respondents admitted to prescribing an antibiotic to a patient because it was easier than explaining the contraindications and 12% gave an antibiotic prescription out of fear of losing good contact with the patient. At the same time, over 76% of participants never offered materials regarding antibiotic or antimicrobial resistance to their patients—reasons not stated. According to the ECDC/PHE report, the most common causes for not dispensing educational resources by prescribers are the lack of valid materials (18%), short visit time (14%) and no interest from the patient (12%) [14].

It stands out that doctors often feel challenged and unprepared to perform adamant yet amicable in-office communication about antibiotics. Miller et al. recommend focusing in patient–doctor conversations on the individual risks (e.g., side effects, potential impact on gut microbiota) rather than the societal charge of AMR when disproving unfounded pressure for antimicrobials [43]. According to Strumann et al., structured communication training offered to physicians based on embracing partnership, empathy and suitable delivery of information on antibiotics proves useful [44]. Clinicians who tune-in with their patients, who are equipped with a better understanding of the psychological factors driving the urge to receive particular medicines and who know the argumentation leverages to hold their treatment strategy were shown to prescribe less antibiotics ambulatorily [45,46,47]. On a more positive note, in a knowledge-gauging study delivered by Mazińska and Hryniewicz, Polish physicians seemed to have a relatively good self-declared comprehension, understanding and application of antimicrobial guidelines in their practice [48]. This resonates with our findings, where over 78% of respondents declared that they prescribed antibiotics “rarely” or ”never” just because they were unsure of the diagnosis. As many as 94% of studied prescribers “rarely” or “never” discontinued antibiotic therapy earlier than necessary. This delivers a positive message about Polish physicians’ preparedness to treat bacterial infections. For comparison, in the mirror study on Jordanian physicians and in the ECDC report, the combined results for “rarely” and “never” in prescribing antibiotics when unsure of diagnosis were 64% and 70%, respectively [14,17]. Continuous effort is needed to educate both health-care workers on pre- and postgraduate levels in communication about AMR as well as the general public in Poland about the risks of the unnecessary use of antibiotics.

Physicians in Poland occasionally have contacts with the pharmaceutical industry and cooperate with companies providing medicinal products [49]. At the same time, doctors are oftentimes approached by pharmaceutical representatives with information on new drugs, including materials with the company’s branding, or through impersonal communication [50,51,52]. Information delivery on a given product is at times accompanied by symbolic gifts which ought to be made in accordance with the national code of ethics for pharmaceutical promotion [53]. Nonetheless, such souvenirs were proven to affect the prescribing practices of doctors, demonstrating favor to the providing company [54,55]. These direct-to-physician advertising actions can concern antibiotics as well, especially those recommended in upper respiratory tract infections [56,57]. Moreover, since the pharmaceutical industry has expanded its advertising effort towards direct-to-patient promotion, it is possible that the “inappropriateness of prescribing antibiotics when they are not necessary” declared by our respondents was partly the industry pressure disguised behind patients’ demands [58,59,60]. There is limited data on interprofessional cooperation between doctors and pharmacists in Poland. According to Zielińska-Tomczak et al., professional contacts mainly focus on correcting prescription errors and do not seem to have an impact on the antibiotic prescription practices of doctors in Poland [61].

### Limitations

The study has a number of limitations. Firstly, the meaning of some questions could have been altered in translation to Polish. However, efforts were made to maintain the meaning and pilot-based adjustments were made to keep the Polish version of the questionnaire in line with the ECDC/PHE original. Another limitation is the usefulness of the original ECDC/PHE questionnaire when it is applied to survey exclusively physicians. However, keeping the original format allowed for easier and interesting comparison with the ECDC/PHE report as well as other studies based on the same model released in the meantime (Jordan, Italy). It is worth noting that the ECDC/PHE survey was made online while in our study the participants filled paper forms. While it might pose a minor comparison bias, the outcomes of our research provide a valuable feed for didactic and policy agents in Poland which was our primary goal. Another limitation is the study group which only consisted of physicians undergoing specialty training accounted for 0.35% of over 150,000 physicians with active license in Poland (2019) [62]. That is why the study group is not representative. Nevertheless, the study group represented different medical specialties, health care institutions and regions from all over Poland. What is more, the knowledge and attitudes of this group of doctors are important, as they indicate the possible directions of changes in the system of postgraduate medical education. It would make sense to carry out an in-depth study among doctors in fields such as family medicine, internal medicine or pediatrics, i.e., those who prescribe antibiotics to their patients relatively frequently.

## 5. Conclusions

The physicians in Poland who participated in this study demonstrated satisfactory knowledge about antimicrobial use, the clinical application of antimicrobial guidelines and the prevention of antibiotic resistance in their respective fields. However, educational interventions for prescribers are needed to help lead challenging communication with patients expecting antibiotics while maintaining unstrained relations. This could be strengthened by equipping doctors with approachable resources for patients on antibiotics and antimicrobial resistance. The One Health approach to the handling of antibiotics as well as hand hygiene in the context of antimicrobial resistance are areas of knowledge which should be highlighted more in continuous medical education for doctors in Poland.

The outcomes of this study can be used to address medical education gaps in under- and postgraduate settings as well as to inform creators of information campaigns on AMR targeting prescribers in Poland. It would be optimal to carry out further studies among doctors of specialties which most often prescribe antibiotics in order to explore the topic in more depth.

## Figures and Tables

**Table 1:** Characteristics of the study group.

Variable, Parameter	Category or Unit	Total (*N* = 504)
Gender, *n* (%)	female	327 (64.88)
	male	170 (33.73)
	no data	7 (1.39)
Age, min–max, M ± SD	year	23–59, 32.8 ± 5.9
Age groups, *n* (%)	23–29	150 (29.76)
	30–39	292 (57.94)
	40–49	37 (7.34)
	50–59	16 (3.17)
	no data	9 (1.79)
Place of residence, *n* (%)	village	7 (1.39)
	town up to 20,000 residents	30 (5.95)
	town 21,000–100,000 residents	83 (16.47)
	town 101,000–200,000 residents	31 (6.15)
	town 201,000–500,000 residents	74 (14.68)
	city over 500,000 residents	272 (53.97)
	no data	7 (1.39)
Length of work as a medical doctor, min–max, M ± SD	year	0–39, 5.1 ± 5.8
Length of work as a medical doctor, years, groups, *n* (%)	1–3 years	290 (57.54)
	4–7 years	104 (20.63)
	8+ years	87 (17.26)
	no data	23 (4.56)
Main place of work, *n* (%)	clinical hospital	201 (39.88)
	public hospital	212 (42.06)
	non-public hospital	7 (1.39)
	public outpatient clinic	22 (4.37)
	non-public outpatient clinic	23 (4.56)
	private practice	6 (1.19)
	other	28 (5.56)
	no data	5 (0.99)
Specialization status, *n* (%)	completed	75 (14.88)
	ongoing	406 (80.56)
	no data	23 (4.56)
Type of specialization, *n* (%)	surgical	79 (15.67)
	non-surgical	402 (79.76)
	no data	23 (4.56)

M—mean, SD—standard deviation.

**Table 2:** Respondents who answered each antibiotic knowledge question correctly.

Key Knowledge Question	✓ Correct Answer − Incorrect Answer	Total (*N* = 504)
Antibiotics are effective against viruses.	−	503 (99.80)
Antibiotics are effective against cold and flu.	−	502 (99.60)
Unnecessary use of antibiotics makes them become ineffective.	✓	492 (97.62)
Receiving antibiotics has associated side effects or risks such as diarrhea, colitis, allergy.	✓	504 (100.00)
Every person treated with antibiotics is at an increased risk of antibiotic-resistant infection.	✓	453 (89.88)
Antibiotic-resistant bacteria can spread from person to person.	✓	478 (94.84)
Healthy people can carry antibiotic-resistant bacteria.	✓	492 (97.62)
The use of antibiotics to stimulate growth in farm animals is legal in the EU.	−	237 (47.02)
The phenomenon of drug resistance in the context of infectious diseases only occurs with bacteria (not, e.g., viruses).	−	408 (80.95)

Results are presented as number and percentage of correct answers, *n* (%).

## Data Availability

The data presented in this study are available on request from the corresponding author Wojciech Stefan Zgliczyński. The data are not publicly available due to privacy restrictions.
